# Palmar-plantar Erythrodysesthesia with Genital Involvement Secondary to Capecitabine Chemoradiotherapy: A Case Report

**DOI:** 10.7759/cureus.3704

**Published:** 2018-12-08

**Authors:** Hsin-pei Hu, Mark T Corkum, Francisco Perera

**Affiliations:** 1 Radiation Oncology, London Regional Cancer Program, Western University, London, CAN

**Keywords:** capecitabine, hand-foot syndrome, palmar-plantar erythrodysesthesia, adverse reaction, toxicity, genital, penis, scrotum

## Abstract

Palmar-plantar erythrodysesthesia (PPE) is a common dermatologic adverse reaction secondary to capecitabine use, but the skin toxicity rarely involves the genitals. We describe a case of PPE with scrotal and penile involvement secondary to capecitabine chemotherapy concurrent with radiotherapy. The patient presented with pain and erythema involving the penis and scrotum during the fifth week of neoadjuvant chemoradiotherapy with capecitabine for T3c N2b M0 low rectal adenocarcinoma. The onset and severity of symptoms in the genitals were loosely associated with the symptoms in the hands and feet. The pain and erythema were self-limiting and improved 11 days after capecitabine discontinuation and local supportive care.

## Introduction

Capecitabine is a prodrug of 5-fluorouracil (5-FU) and a chemotherapeutic agent used for colorectal cancers as adjuvant or neoadjuvant therapy, with or without radiation, or as first-line treatment for metastatic cancer. Palmar-plantar erythrodysesthesia (PPE) is the most common dermatologic adverse reaction associated with both capecitabine and 5-fluorouracil (5-FU). A painful erythematous rash secondary to capecitabine typically localizes to the palms, fingers, and feet. Capecitabine rarely causes PPE with genital involvement; few reported cases have described PPE with genital involvement during capecitabine chemoradiotherapy [1-3]. We present a case of PPE with scrotal and penile involvement secondary to concurrent capecitabine chemotherapy and radiotherapy.

## Case presentation

An otherwise healthy, 43-year-old Caucasian man was diagnosed with clinical/radiologic T3c N2b M0 low rectal adenocarcinoma with a threatened circumferential resection margin. His complete blood count, renal, and liver function tests were within normal limits. The patient underwent dihydropyrimidine dehydrogenase (DPYD) genotype testing as part of a personalized medicine program, which suggested normal DPYD enzymatic activity. He started neoadjuvant chemoradiation therapy consisting of standard-dose capecitabine (825 mg/m^2^ oral twice daily, seven days a week) and 5040 cGy in 28 fractions with a concurrent boost to 5760 cGy in 28 fractions to the primary tumor and involved lymph nodes delivered by tomotherapy. After 23 fractions, the patient presented with pain and erythema involving his hands and feet as well as his penis and scrotum. The patient also reported narrowing and deviation in the direction of his urinary stream due to a white exudate, which had developed at the urethral opening. The patient had self-limiting, sharp cramping pains in the mid-abdomen. He was instructed to discontinue capecitabine. Radiotherapy was placed on hold.

**Figure 1:**
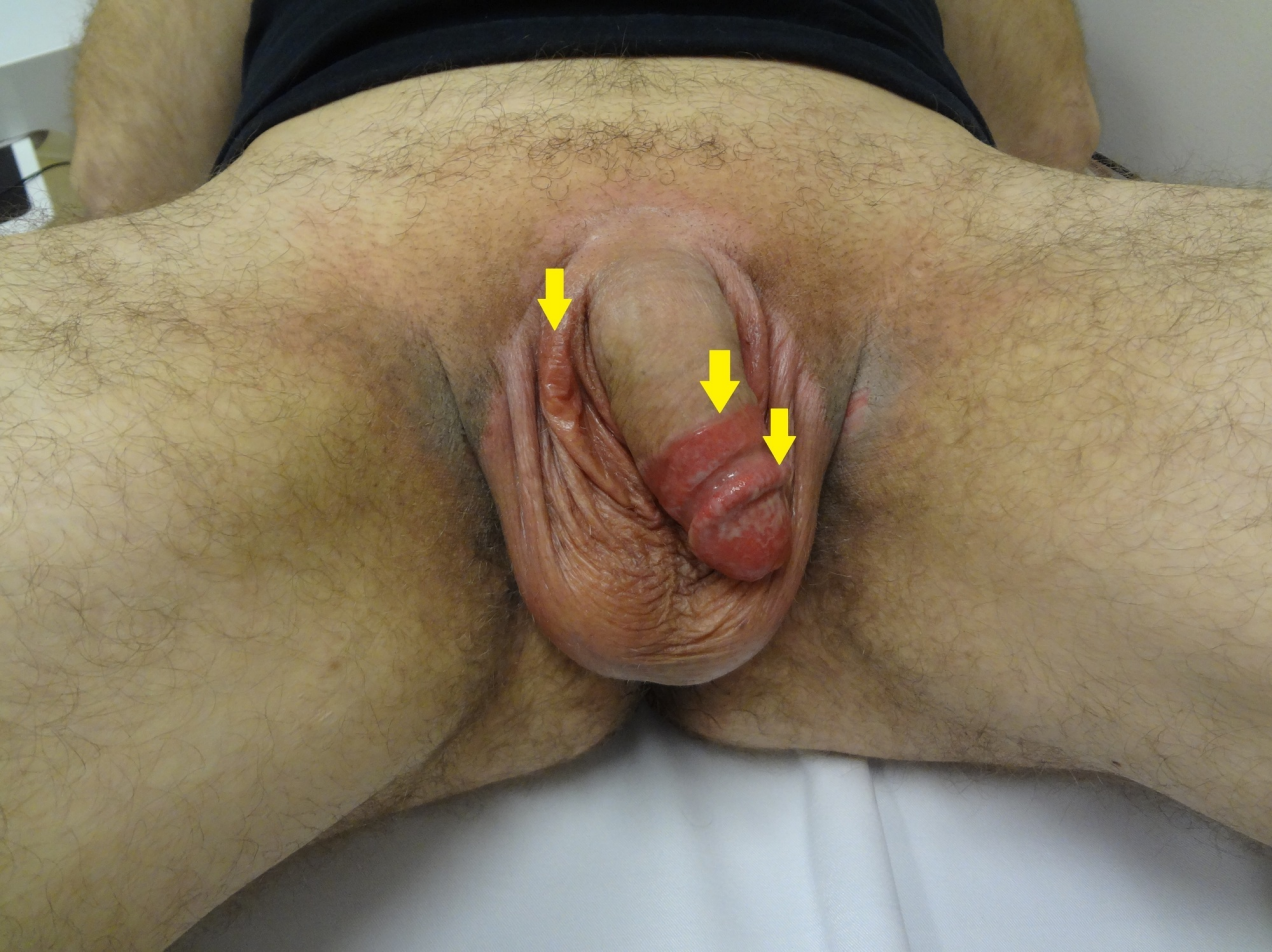
Circumferential erythema to the glans penis and mild erythema in the skin folds of the scrotum (arrows)

**Figure 2:**
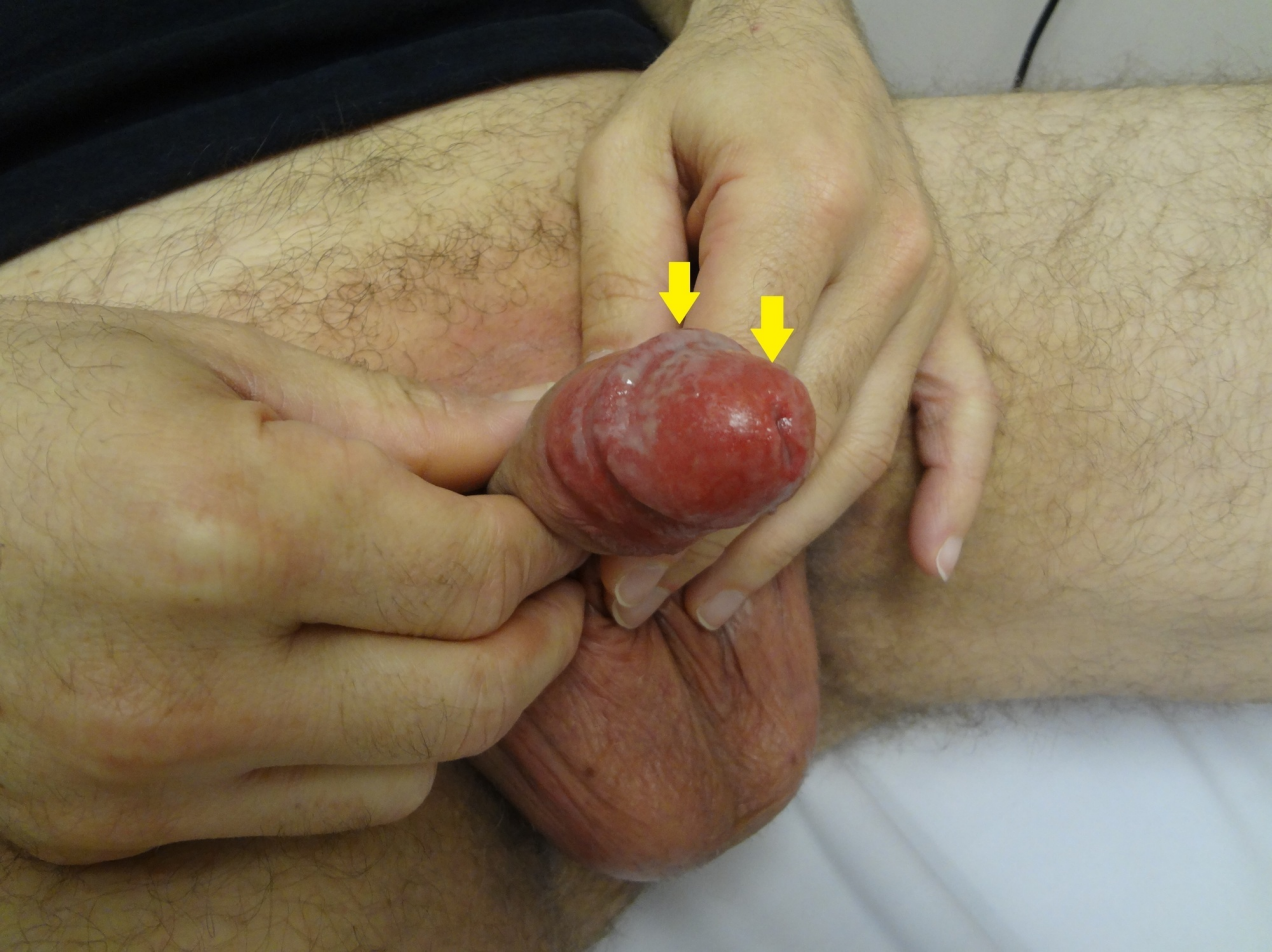
Thin, white exudate covering the proximal glans penis (arrows)

Five days after discontinuing capecitabine, the pain and redness in his hands and feet had improved, but the findings at the tip of his penis had not improved. On physical examination, there was circumferential erythema to the glans penis (Figure 1). There was a thin, white exudate affecting the corona and tip of the glans (Figure 2). The groin and lateral aspects of the scrotum had mild erythema in the skin folds. In the perineum and perianal areas, there was more extensive erythema with non-confluent moist desquamation with exudate consistent with radiation dermatitis. The patient was instructed to apply petroleum jelly and non-stick gauze pads to the affected area and increase water intake. The exudate demonstrated probable contamination with Pseudomonas aeruginosa. No antibiotics were started. Seven days after discontinuing capecitabine, the penile erythema and exudate, as well as the perianal moist desquamation, had begun to resolve. Eleven days after discontinuing capecitabine, improvement in pain, erythema, and urinary symptoms were noted. The patient underwent the remaining fractions of radiotherapy without capecitabine. One-month post-treatment magnetic resonance (MRI) showed a decrease in tumor volume with a plan to undergo a low anterior resection of his rectal cancer.

## Discussion

The differential diagnoses for the genitourinary symptoms in our patient include allergic reaction, contact dermatitis, radiation dermatitis, and PPE-like phenomenon associated with capecitabine. Post hoc analysis of the radiation treatment plan (in 28 fractions) demonstrated Dmax of 21.4 Gy and Dmean of 14.2Gy to the penis and Dmax of 23.3 Gy and Dmean of 9.2 Gy to the scrotum, doses unlikely to cause such an acute, severe reaction. PPE with genital involvement secondary to capecitabine use is the likely cause considering the relevant symptoms in the hands and feet, the symptom onset coinciding with the capecitabine regimen, the external genitalia most affected distally (acrodermatitis), and the subsequent self-limiting resolution following discontinuation.

Capecitabine is enzymatically converted to its active form, 5-FU, which acts as an antimetabolite that disrupts DNA replication. Toxicity often disrupts the hematologic, gastrointestinal, and dermatologic systems and severe reactions may necessitate treatment discontinuation [4]. DPD accounts for more than 80% of 5-fluorouracil metabolism and reduced or deficient DPD enzymatic activity predisposes patients to more grade 3-4 adverse reactions [5-6]. Genotype testing in our patient tested the DPYD gene for three common translocations accounting for reduced or deficient DPD activity (IVS14+1 G>A, DPYD 2846 A>T, and DPYD 1679T>G), which were normal [7]. Despite his normal DPYD genotype testing, our patient experienced moderate gastrointestinal side effects as well as severe dermatologic toxicity.

PPE is a common adverse reaction in patients undergoing capecitabine treatment. PPE is characterized by erythema, discomfort, peeling, and cracking of the hands or feet. PPE secondary to capecitabine use typically occurs within the first three cycles of chemotherapy [8]. National Cancer Institute grades PPE toxicity from grade 1 to 3, where each higher grade represents more pain, more prominent skin changes, and higher impairment to activities of daily living [9]. Our patient developed grade 2 toxicity in the hands and feet: painful erythema that affects the activities of daily living. There is currently no grading system to describe PPE manifestation in the genitalia. PPE involving the genitals has been proposed as a grade 4 toxicity given that its presentation in the affected area tends to parallel grade 3 toxicity [1]. However, our patient’s grade-2-like toxicity in the genitalia suggests further subclassification may be appropriate.

Chemotherapy-induced PPE rarely involves the genitals. Our case report is the first reported reaction during neoadjuvant chemoradiation for rectal cancer. Our literature review found a few cases of capecitabine-associated PPE presenting with painful erythema with swelling and ulceration of the scrotum and penis (Table 1) [1-3]. Its active form, 5-FU, has also rarely shown skin toxicity with penile or scrotal involvement [10-11]. Tyrosine kinase inhibitors and liposomal anthracyclines are other drugs that rarely cause blistering or ulcerating lesions on the penis or scrotum with concurrent skin toxicity in the hands and feet [12-13]. In many cases, hand and/or foot manifestations typically coincide with the symptoms in the genitals, but there are loose associations in onset, severity, and symptom resolution.

**Table 1:** Summary of reported patient cases of PPE with genital involvement secondary to capecitabine use PPE: Palmar-plantar erythrodysesthesia

Author	Age	Sex	Race	Primary malignancy	Chemotherapy regimen	?Radiotherapy	Symptom onset	Symptom treatment	Resolution	? Recurrence with additional chemotherapy
Present Case	43	M	White	Rectal	825 mg/m^2 bid	Yes	4th wk	Discontinuation	11 days	-
Sapp and DeSimone [1]	67	M	White	Colon	Capecitabine (1000 mg/m^2 bid x 14 d w/2 wk rest)	No	3rd cycle	Discontinuation	2 wks	-
	63	M	White	Colon and gastric	Capecitabine (1000 mg/m^2 bid x 14 d w/ 1 wk rest), then oxaliplatin (100 mg/m^2 x?d)	No	1st cycle (day 12)	Discontinuation	2 wks	-
Fleta-Asín et al. [2]	84	M	?	Colon	Capecitabine (?)	No	?	Discontinuation	10 days	Yes
	78	M	?	Colon	Capecitabine (?)	No	After 1st cycle	Discontinuation	2-3 wks	Yes
	73	M	?	Rectosigmoid	Capecitabine (?)	Yes	2nd wk	Topical corticosteroids	2 wks	No
Ljubojevic et al. [3]	63	M	?	Caecum	Capecitabine (?) and irinotecan (?) + bevacizumab (?)	No	After 1st cycle	Discontinuation	3 wks	Yes

The mainstay treatment for PPE is a modification or interruption of the offending drug agent as well as pain management and supportive care [14]. Systemic corticosteroids and pyridoxine show varying degrees of efficacies in treating PPE [14-15]. Local supportive measures, such as cooling the affected areas, can lessen drug extravasation into the local tissues and maybe a preventative strategy for PPE [16]. Moisturizing the areas of ulceration helps maintain skin integrity and improve healing [17]. Topical antioxidants and antiperspirants may help in preventing and reducing the severity of PPE [18-19]. Symptoms typically resolve within two to four weeks.

## Conclusions

We present a rare case of PPE with genital involvement associated with capecitabine chemotherapy and concurrent radiotherapy. Recognizing that PPE secondary to capecitabine use can occur outside the characteristic locations in the hands and feet is the key to swift management of patient discomfort and potentially harmful complications.
